# COVID‐19 infection as a possible cause of complete heart block: What do we know so far?

**DOI:** 10.1111/anec.12930

**Published:** 2022-01-24

**Authors:** Anis Abobaker

**Affiliations:** ^1^ Rehabilitation and Care of Elderly Mid Cheshire Hospitals NHS foundation trust Crewe UK

## CONFLICT OF INTEREST

I declare that I have no conflict of interest.

Dear Editor,

There are many reports in the literature that documented the association between COIVD‐19 infection and development of brady‐arrhythmias and complete heart block (CHB). My aim in this letter is to summarize the possible causes of CHB in patients with recent COVID‐19 infection. One of the possible causes of CHB in COVID‐19 patients is myocarditis. An elderly female with a diagnosis of COVID‐19 myocarditis, evident by significantly raised troponin and absence of regional wall abnormalities on echocardiogram, developed transient CHB which required temporary transvenous pacing (Mohamed, 2021). Other patients with fulminant myocarditis associated with severe left ventricular dysfunction secondary to COVID‐19 infection developed CHB which resolved following the treatment of the infection and improvement of inflammatory markers (El‐Assaad et al., 2020; Nikoo et al., 2021). In fact, persistent CHB, which required permanent pacemaker (PPM) insertion, has been reported in a middle‐aged male patient who developed subclinical myocarditis following COVID‐19 infection (Al‐assaf et al., 2020). This patient had normal inflammatory markers and normal cardiac enzymes, and the echocardiogram was unremarkable. The diagnosis of myocarditis was made by cardiac MRI which showed edema of the interventricular septum. The other possible explanation of the incidence of CHB in the setting of COVID‐19 infection is direct invasion and damage of cardiac conduction system by SARS‐CoV‐2 virus. A 35‐year‐old male patient without a previous past medical history presented with CHB 4 days after the onset of COVID‐19 infection (Alabdulgader et al., 2020). Interestingly, this patient developed a cardiac conduction‐specific illness. In addition to CHB, he developed sinus atrial node dysfunction and intraventricular conduction block, in absence of myocarditis or coronary obstruction. This patient had persistent viremia evident by 3 consecutive PCR tests, which could be the trigger for direct cardiac invasion and damage by the virus.

**FIGURE 1:**
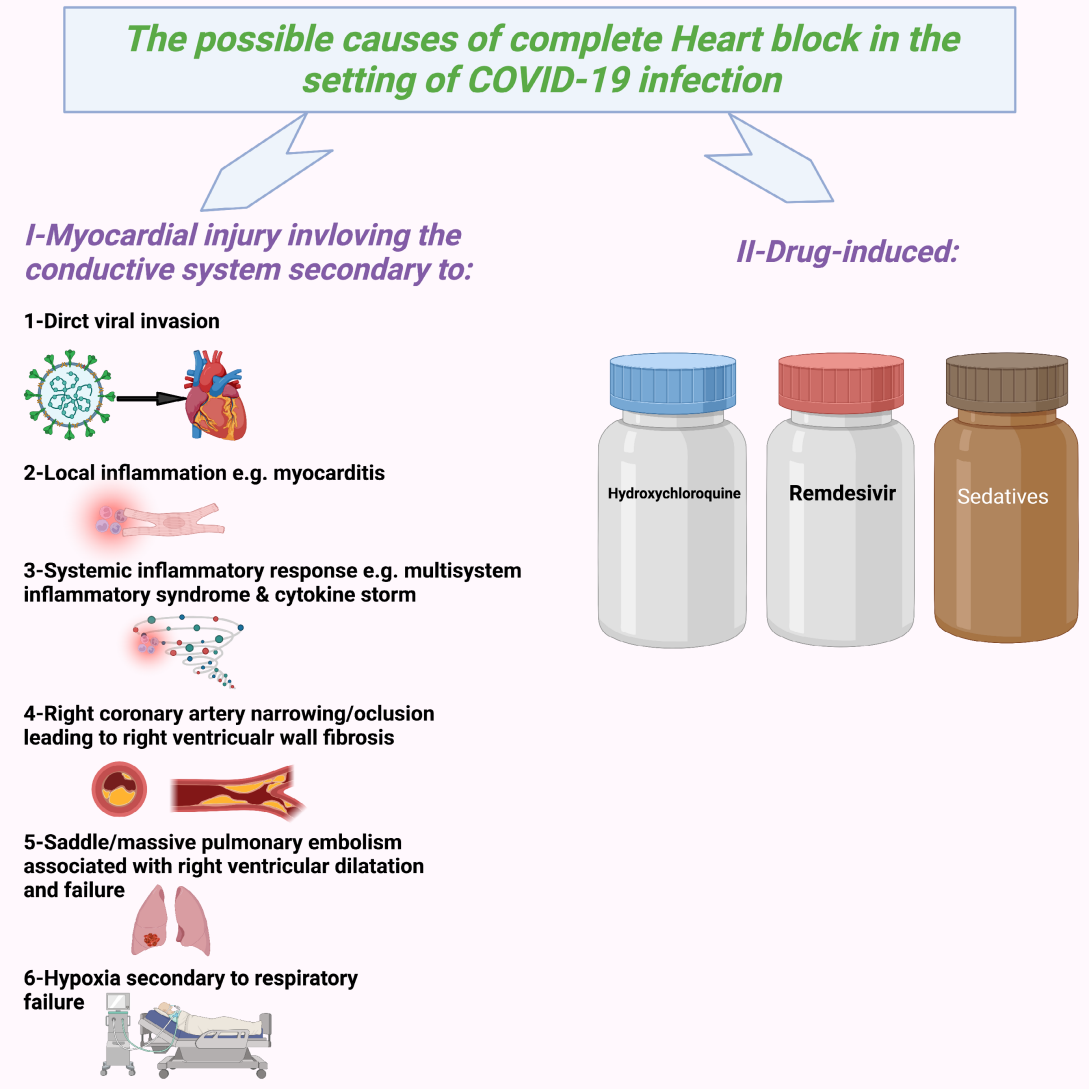
The possible causes of complete heart block in the setting of COVID‐19 infection (created with biorender.com)

Other causes of cardiac injury with subsequent development of CHB in patients with COVID‐19 have been reported (Figure 1). Cardiac injury leading to temporary CHB could happen as a consequence of pediatrics multisystem inflammatory syndrome (Kawasaki‐like reaction) in patients with COVID‐19 (Mehta et al., 2021). In addition, myocardial injury and CHB following COVID‐19 has been attributed to systemic inflammatory response, such as cytokine storm (Gyawali et al., 2021). An 8‐year‐old boy with confirmed COVID‐19 infection presented with features of right‐sided heart failure and bradycardia (Sisko et al., 2021). Electrocardiogram confirmed the diagnosis of CHB, and echocardiogram showed severe right ventricular failure, with normal left ventricular function. In his case, PPM insertion was indicated secondary to persistent CHB. Coronary angiogram showed significant narrowing of right coronary artery, and cardiac MRI showed diffuse fibrosis of the right ventricular wall, but no features of myocarditis. A 52‐year‐old male patient without a significant past medical history presented with COVID‐19 pneumonia complicated by saddle pulmonary embolism and right ventricular dilation leading to transient CHB, which was resolved after treatment with heparin (Ismail et al., 2021). A case series reported the incidence of CHB in 3 critically‐ill COVID‐19 patients with hypoxic respiratory failure who required mechanical ventilation (Ahmad et al., 2021). The possible causes of CHB in these patients could be,hypoxic damage to the myocardium, sedative use, and metabolic derangement.

It is argued that CHB in patients with COVID‐19 infection could be drug‐induced. A young female patient presented with a brief episode of loss of conscious, chest pain, and itchy skin rash three weeks following COVID‐19 infection (Firouzabadi et al., 2020). Cardiac monitoring showed a transient CHB which reverted spontaneously to sinus rhythm. During the acute infection, the patient was treated with hydroxychloroquine, which could be the trigger for CHB, as it leads to fascicular block as a side effect which might progress to a high degree atrio‐ventricular block. Interestingly, CHB was reported after initiation of remdesivir for treatment of COVID‐19 infection, and resolved spontaneously two days after the treatment was discontinued (Selvaraj et al., 2021). The timing of development and resolution of the heart block raised the suspicion of drug‐induced CHB.

In conclusion, CHB might occur following COVID‐19 infection as a complication of myocardial injury or a side effect of the drugs which were used during the management of COVID‐19 infection. In most cases, CHB was transient, and resolved spontaneously or after successful treatment of COVID‐19 infection. However, cases of persistent CHB, as a complication of COVID‐19 infection, which required the insertion of PPM have been reported.

## Data Availability

Data sharing not applicable to this article as no datasets were generated or analysed during the current study
